# Supplementary material for: A Critical View of the Application of the APEX Software (Aqueous Photochemistry of Environmentally-Occurring Xenobiotics) to Predict Photoreaction Kinetics in Surface Freshwaters
Source: Molecules. 2019 Dec 18;25(1):9. doi: 10.3390/molecules25010009 (PMC7017383; doi:10.3390/molecules25010009)
Supplement: Supplementary file 1 [file molecules-25-00009-s001.zip › 3.2.4_gcc-4.4.0.zip!/3.2.4_gcc-4.4.0/3.2.4_gcc-4.4.0/doc/octave/html/interpreter/Test-Plotting-Functions.html]

Test Plotting Functions - Untitled


Previous: Interacting with plots,
Up: Plotting Basics


---

#### 15.1.8 Test Plotting Functions

The functions `sombrero` and `peaks` provide a way to check
that plotting is working. Typing either `sombrero` or `peaks`
at the Octave prompt should display a three-dimensional plot.

— Function File: **sombrero** (n)  
> Produce the familiar three-dimensional sombrero plot using n
> grid lines. If n is omitted, a value of 41 is assumed.
>
> The function plotted is
>
> ```
>           z = sin (sqrt (x^2 + y^2)) / (sqrt (x^2 + y^2))
> ```
>
>
>
>
> **See also:** surf, meshgrid, mesh.

— Function File: **peaks** ()  
— Function File: **peaks** (n)  
— Function File: **peaks** (x, y)  
— Function File: z = **peaks** (...)  
— Function File: [x, y, z] = **peaks** (...)  
> Generate a function with lots of local maxima and minima. The function
> has the form
>
> ```
>      f(x,y) = 3*(1-x)^2*exp(-x^2 - (y+1)^2) ...
>               - 10*(x/5 - x^3 - y^5)*exp(-x^2-y^2) ...
>               - 1/3*exp(-(x+1)^2 - y^2)
> ```
>
> Called without a return argument, `peaks` plots the surface of the
> above function using `mesh`. If n is a scalar, the `peaks`
> returns the values of the above function on a n-by-n mesh over
> the range `[-3,3]`. The default value for n is 49.
>
> If n is a vector, then it represents the x and y values
> of the grid on which to calculate the above function. The x and
> y values can be specified separately.
>
>
>
> **See also:** surf, mesh, meshgrid.
